# Environmental health determinants among seasonal migrants in China: a multi-dimensional analysis

**DOI:** 10.3389/ijph.2026.1610170

**Published:** 2026-10-22

**Authors:** Wanqing Zheng, Sara Akram

**Affiliations:** 1 School of Marxism, Sun Yat-sen University, Guangzhou, China; 2 Center for Studies of Hong Kong, Macao and Pearl River Delta, Sun Yat-sen University, Guangzhou, China; 3 National Business School, The University of Faisalabad, Faisalabad, Pakistan

**Keywords:** agricultural workers, air pollution, China, climate change, environmental health

## Abstract

**Objective:**

To conduct a systematic review of the evidence regarding the impact of environmental health factors, such as air pollution, thermal stress, water and soil pollution, agricultural instability, and occupational hazards, on seasonal migration in China.

**Methods:**

A systematic review of PubMed, Web of Science, CNKI, Scopus, and Google Scholar (2001–2025) was conducted to identify peer-reviewed articles and institutional reports on environmental exposure, health, and migration outcomes in the Chinese mainland’s populations.

**Results:**

Outmigration trends were found to be associated with each of the four aspects of air pollution, heat stress, water contamination, and agricultural instability during the winter, and, in addition to the above, water and soil contamination, in the summer. The effects were found to be strongest among rural poor and older individuals. At urban destinations, migrants were also at risk of occupational hazards due to silica dust and chemicals.

**Conclusion:**

The environment exerts a multidimensional, seasonal influence on internal migration in China. There is a need for coordinated environmental health governance to minimize migrant exposure and promote health equity in this population.

## Introduction

China has the largest internal migration system in the world. By 2022, internal migrants [1] represented a floating population (internal migrants living outside their place of household registration) that had increased to more than 380 million, approximately 27% of the inhabitants, up more than a factor of 30 since 1980 (Figure 1) [1, 2]. Many migrants come and go on an annual or cyclical basis from their rural homes to urban destinations. Previous research on the wage gap between coastal and inland regions has suggested that this is the main factor that influences migration; however, increasing evidence suggests that environmental health factors also have a direct influence on migration, independent of the wage differential [3, 4]. This is supported by studies of urban growth patterns, industrial pollution siting, environmental policy and trade, and pediatric environmental burden [5–8]. Winter air quality often exceeds the World Health Organization (WHO) safety standards by more than twelvefold in many northern cities [9]; groundwater and soil are highly contaminated with heavy metals in many rural regions [10, 11]; and heat waves, floods, and droughts threaten crop production, causing heat stress for agricultural workers [12, 13].

**FIGURE 1:**
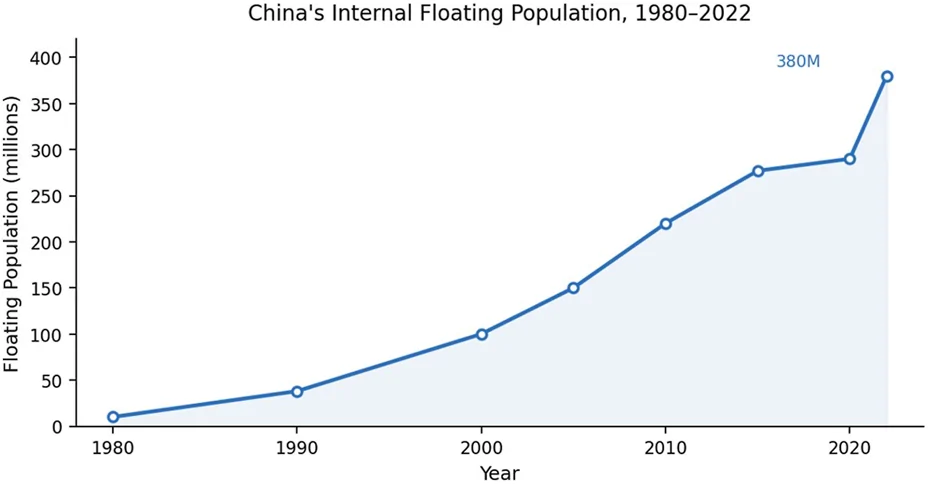
Growth of China’s internal floating population, 1980–2022. Systematic review, China, 1980–2022.

Migration is influenced by environmental conditions in two connected ways: through environmental degradation affecting health and therefore labor productivity (direct pathway), and through environmental degradation affecting rural livelihoods and resulting in an economic push to migrate (indirect pathway) [2]. In China, seasonal migration tends to follow the same patterns as environmental stress, such as air pollution in winter, heat in summer, and pesticide use in spring.

The objectives of this review are to outline the main environmental health stressors to which migrants are exposed in China before and after migration; to summarize health outcomes related to these stressors; and finally, to highlight gaps in the research and policy implications. Using terminology from the literature, the floating population is defined as all the individuals who live outside their household registration (hukou) area; the seasonal or cyclical migrants are a subset of the floating population who move and return once a year, typically in response to a particular seasonal, climatic, or construction-related event; and environmental migrants are those whose moves are significantly influenced by exposure to the environment and the resulting impact on their livelihoods. The annual migration of older individuals to Hainan for climate and health reasons has been well documented in China as an example of environmentally initiated seasonal migration [14–18], and is representative of the pattern of seasonal migration that is the subject of this review at the national scale.

Previous studies have reviewed the relationship between climate migration in general [4, 19, 20], occupational hazards [21], and water and soil pollution [10]. However, no previous studies have considered air quality, thermal stress, water pollution, soil pollution, and agricultural instability within a single framework, or have tied these stressors to the seasons of migration. In this review, these gaps are addressed by integrating five environmental domains into one analytical framework and mapping their seasonal timing (see Results). The broader theoretical framework on climate vulnerability migration, transnational patterns of climate-driven migration, and decision-making on migration in the absence of certainty also supports the approach followed here [22–24].

## Methods

### Literature search and eligibility criteria

A systematic search was carried out in PubMed, Web of Science, China National Knowledge Infrastructure (CNKI), Scopus, and Google Scholar between 1 January 2025 and 30 May 2025, using terms related to internal migration (e.g., “floating population,” “hukou,” “rural-to-urban migration,” “seasonal migration”), environmental exposure (e.g., “air pollution,” “PM2.5,” “heat stress,” “water contamination,” “soil pollution,” “pesticide exposure”) and health outcomes (e.g., “respiratory disease,” “occupational injury,” “chronic kidney disease,” “mortality”). A manual search of the reference lists of the retrieved studies was undertaken to identify any further relevant studies.

Studies were eligible if the study population lived in mainland China; at least one environmental exposure was quantified (e.g., exposure to PM2.5 that exceeded the World Health Organization guidelines, or temperatures above 35 °C, or concentrations of heavy metals above Chinese national safety standards); at least one health or migration-related outcome was reported (e.g., mortality, disease incidence, biomarker levels, out-migration rate, or timing of seasonal departure); and the study was published in a peer-reviewed journal or official government report between 1 January 2001 and 30 May 2025. Studies of laboratory animals, or *in vitro* studies; studies lacking original data (e.g., reviews, editorials, or conference abstracts); studies conducted in populations other than mainland China (such as international migrants or non-mainland populations); and articles not available in full text were not included.

### Study selection

Approximately 1,200 records were retrieved in the first database search. After full-text review, 75 studies were excluded (no original data, non-mainland population, or no clear health or migration outcome) and 75 met the eligibility criteria due to a lack of original data, a non-mainland Chinese population, or a lack of a clear health or migration outcome. Forty-one more sources were found by manually searching the reference lists giving a total of 116 included studies and reports (75 + 41). Finally, after a synthesis guided by the PRISMA 2020 reporting guidelines [25], 116 peer-reviewed studies and institutional reports published between 2001 and 2025 were included (Figure 2). This systematic review was not prospectively registered.

**FIGURE 2:**
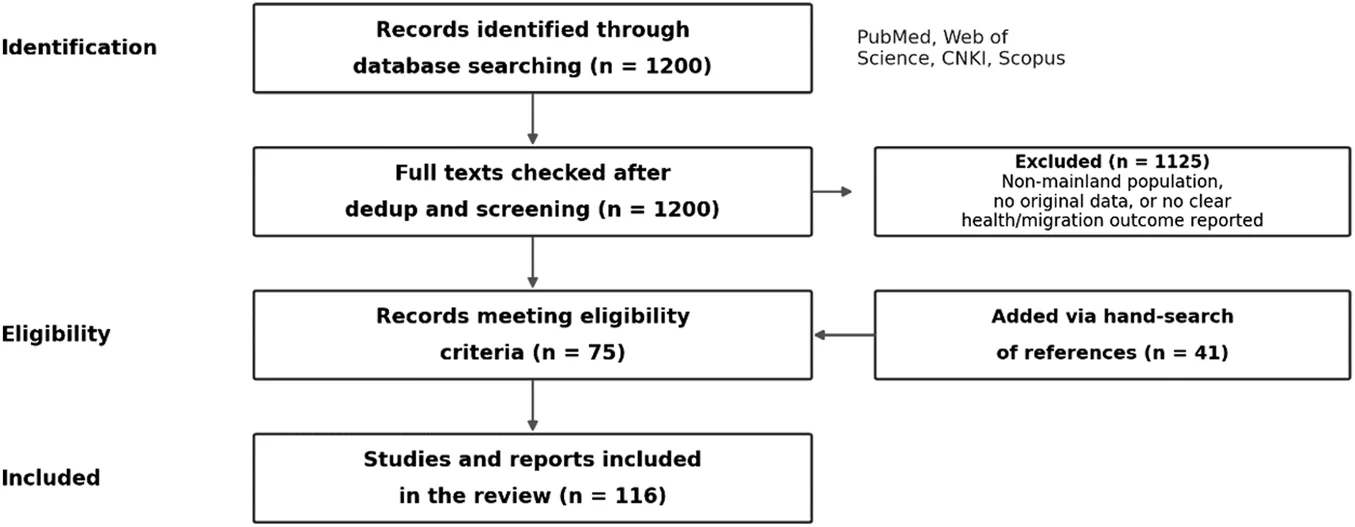
PRISMA 2020 flow diagram of study identification, screening, eligibility assessment, and inclusion. Systematic review, China, 2001–2025.

### Quality appraisal

A modified version of the Newcastle-Ottawa Scale (NOS) [26] was used for the appraisal of the quality of the articles, which included cohort and cross-sectional studies. We also used the JBI Critical Appraisal Checklist for descriptive studies [27, 28] for three domains: selection of participants; comparability of groups; and quality of outcome measurement. No study was excluded due to quality alone, and higher-quality studies were given more weight in the qualitative synthesis. A formal risk-of-bias assessment was not conducted, as no single standardized instrument could be used uniformly across the 116 studies included, due to the epidemiological cohorts, household surveys, and government reports included having a variety of study designs and outcome definitions (see Limitations).

### Analytical framework

The synthesis is structured around five areas of environmental health: air quality, thermal stress, water and soil contamination, agricultural instability, and occupational hazards at migration destinations. A temporal perspective was used to explore each domain by separating exposures experienced in rural places from exposures in urban destinations, thereby understanding how migration itself can buffer or mediate environmental health risks. This was followed by a cross-cutting analysis on the influence of gender, age, and socioeconomic status on exposure and mobility in each of the five domains.

In the Results section, direct evidence refers to studies that measure the behavior of migration itself, while indirect evidence refers to studies that measure the environmental impact on health, agriculture, or income, but not migration. When evidence was indirect, causal language was avoided, and terms such as “associated with,” “may contribute to,” or “may affect migration decisions” were used to clearly indicate the strength of the underlying evidence to the reader.

## Results

### Historical trajectory: environment and migration, 1980–2022

The relationship between environment and migration in China can be divided into three general stages. Stage 1 (1980-2000): first wave of rural-to-urban migration, industrialization and coastal pollution. Stage 2 (2001-2015): rapid growth of the floating population and pollution spreading inland; Jan 2013 PM2.5 episode and 2014 soil survey as turning points. Stage 3 (since 2016): improving urban air quality alongside rising thermal stress, water scarcity and climate pressure on agriculture. End the paragraph by stating that air-quality gains have been accompanied by growing pressure from other stressors. The first wave of rural-to-urban migration, which more than doubled the floating population between 1980 and 2000, from under 10 million to approximately 100 million, was accompanied by rapid industrialization and worsening air and water pollution in coastal regions, with the Yellow River drying up in 1997. Pollution sources and exposure shifted geographically from coastal to inland regions between 2001 and 2015, while the floating population increased by more than 250 million, with the PM2.5 concentration in Beijing reaching 900 μg per cubic meter in January 2013: this was a turning point in raising awareness at the public and policy levels. In the 2014 national soil survey, 16.1% of the farmland failed quality standards. Urban air quality has improved significantly since 2016, as PM2.5 levels dropped 30%–35% across major cities from 2014 to 2020 under the War on Pollution. Meanwhile, thermal stress and water scarcity have increased, especially in the central and western provinces, and are now significant drivers of migration, as is the impact of climate disruption on agriculture, which has been an increasing force particularly since 2018. As of May 2022, the floating population was more than 380 million. This trajectory indicates that as one environmental stressor (air pollution) is managed through specific policy, other stressors tend to emerge or intensify, although not necessarily to the same degree. (“The path” = the historical trajectory described above.)

### Air quality as a driver of seasonal migration

Some of the most well-documented environmental health risks in China include fine particulate matter (PM2.5), nitrogen dioxide, and ground-level ozone. In 2017, approximately 1.24 million deaths in China were attributable to outdoor particulate pollution, or 29% of global deaths [29]. Air pollution is very seasonal, with winter coal heating causing high PM2.5 concentrations (up to 300 μg per cubic meter) in cities such as Beijing, Shijiazhuang, and Harbin, at a level nearly 20 times higher than the WHO 2021 24-h PM2.5 guideline is 15 μg per cubic meter). In the summer, strong sunlight and vehicle exhaust emissions lead to high ozone concentrations [30]. There are approximately 175 million rural households using solid fuels for cooking and heating, which causes indoor air pollution [31], and systematic national exposure datasets support this estimate [32, 33].

Long-term exposure to PM2.5 is linked to an increased risk of cardiovascular disease, stroke, chronic obstructive pulmonary disease, lung cancer, and lower respiratory infections [34–36]. In 272 cities in China, an increase of 10 μg per cubic meter in PM2.5 was associated with a 0.22% increase in daily all-cause mortality [34], and children in high-pollution areas have reduced lung function and increased asthma rates [37]. The estimated impact of indoor solid-fuel use on deaths in 2013 was approximately 900,000 deaths (5.7% of all-cause mortality) [38], and the estimated difference in life expectancy between the north and the south of the country is approximately 1.8 years (Figure 3) [29]. Other Chinese and international cohort studies also provided supporting data on death rates, depression risk, diabetes incidence, and cardiometabolic outcomes due to PM2.5 [39–47].

**FIGURE 3:**
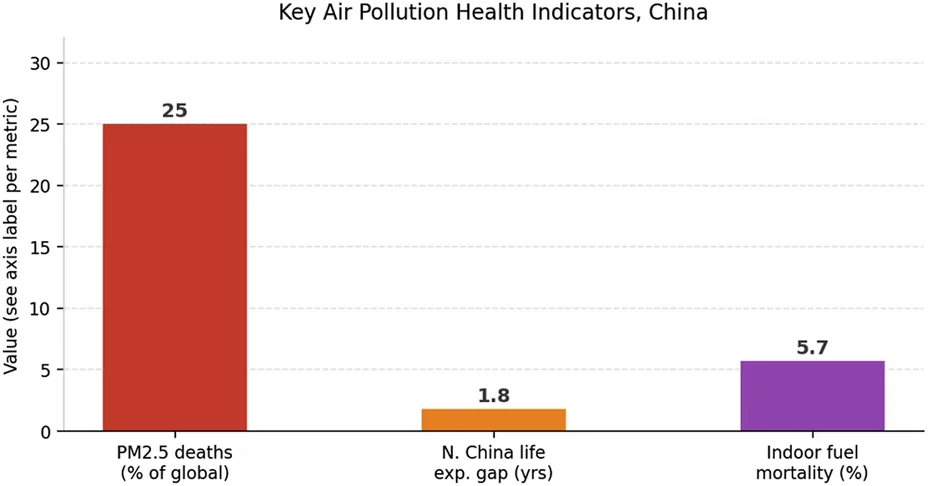
Key air pollution health indicators in China. Systematic review, China, 2001–2025.

There has been an increase in evidence on the direct impact of air pollution on migration decisions since 2015. One study on migration and ambient PM2.5 exposure using the Chinese General Social Survey revealed that higher exposure to PM2.5 in one’s place of origin (PPO) was linked to increased migration, independent of income, and was highest during the winter heating season [3]. In the industrial regions of Hebei Province, qualitative fieldwork revealed that mothers commonly moved temporarily with their children in winter due to respiratory health problems [4]. This air pollution-migration relationship is supported by a large body of empirical literature that shows that air pollution is negatively correlated with settlement intention and/or choice of destination in national surveys, in city-pair studies, and in push-pull frameworks [48–77]. Pollution-driven migration is distinct from labor-market-driven migration in that it is not seasonal but is triggered by air quality episodes. Additionally, this specific type of migration is skewed toward caregivers and children.

### Thermal stress and extreme temperature events

Since 1960, average temperatures in China have increased by 0.22 °C–0.33 °C per decade, which is twice the global average of 0.79 °C per decade [12], and heat wave frequency has increased by approximately 60% over the last 20 years, and the number of heat-related deaths has also increased. By 2050, climate projections indicate that there will be 10–20 days per year with physiologically hazardous outdoor conditions in the major cities of China [78].

Heat exhaustion, heat stroke, dehydration, and the exacerbation of cardiovascular, renal, and respiratory disease occur at extreme temperatures [12, 79]. Outdoor workers are most at risk (especially farmers and construction workers), and rice and wheat farmers in the Yangtze River Delta and in the North China Plain work more than 6 h outdoors each day during peak heat [80], which has been associated with an increased prevalence of chronic kidney disease among agricultural workers [81]. In fact, historically, the number of deaths from cold-related causes in 66 Chinese cities has been higher than the number of deaths from heat-related causes [82]. Similarly, other surveillance evidence has reported a greater number of cold-related deaths compared to heat-related deaths [83] in less well-insulated homes in the central and southern regions of China.

Heat stress has direct (worker health) and indirect (crop yield) impacts on migration. The potential loss of wheat yield is approximately 3%–8% for every 1 °C increase above the optimal temperature occurring during flowering and grain filling. Rice in southern China is similarly sensitive [84]. Households that rely on small-scale farming face pressure to turn to other sources of income when agricultural income drops, and out-migration tends to occur in the period between the harvesting of the previous crop and before planting the next one, which roughly corresponds to the agricultural year [3].

### Water and soil contamination

Nitrate contamination is the most common contaminant in the water, affecting more than 60% of monitored wells in northern China; arsenic [85] is the most common contaminant in drinking water, affecting an estimated 19.6 million individuals, primarily in Inner Mongolia, Shanxi, and Xinjiang [11]. Approximately 14% of river sections and 25% of lake monitoring points did not meet safe contact standards in 2020 [86].

In 2014, a nationwide soil survey revealed that 16.1% of agricultural soils were outside of the acceptable range [87] (Figure 4), mainly due to cadmium in rice in Hunan Province. Cadmium exposure can lead to kidney damage, poor bone density, and is associated with an increased risk of certain cancers [88]. Children living close to e-waste recycling centers in Guangdong and Zhejiang have higher blood lead levels; in Guiyu, the average child’s blood lead level is nearly three times the international guideline of 10 μg per deciliter [89].

**FIGURE 4:**
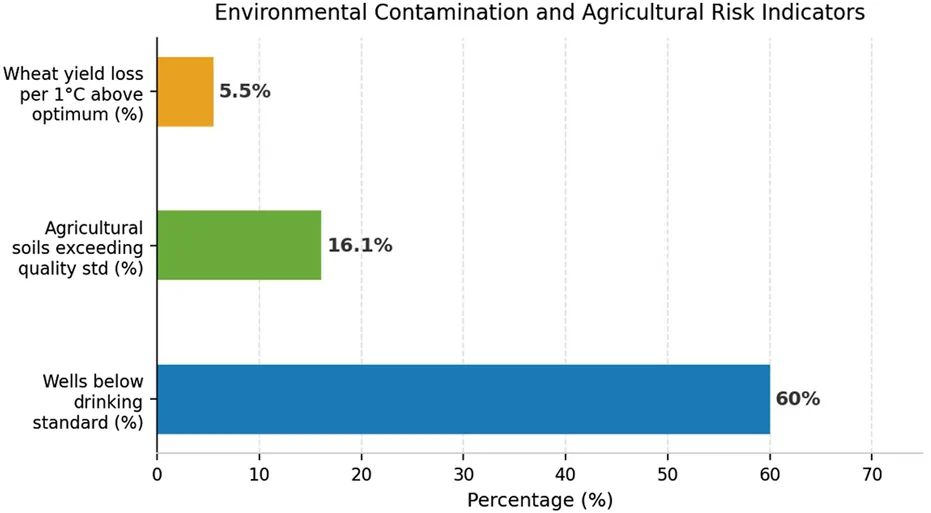
Environmental contamination and agricultural risk indicators for China. Systematic review, China, 2001–2025.

Water scarcity exacerbates such contamination issues, especially in the north and northwest of the country. Over 150 million individuals in the Yellow River basin are increasingly facing water scarcity problems due to overexploitation, reduced glacial melt, and increased evaporation [90]. Household water insecurity has been linked to poor sanitation, diarrhea, decreased food quality, and psychosocial stress, which can encourage outmigration [91]; water shortages before the spring monsoon are an important factor in determining the timing of migration from arid areas [91].

### Climate-related agricultural instability and occupational hazards at migration destinations

Farming systems are becoming more fragile in response to warming and increasing flood and drought frequency, while technological progress has not been able to improve wheat and maize production in northern provinces since the 1980s [84, 92]. The relationship between migration decisions and the variability in rainfall within a single agricultural season appears to be more significant than the relationship with long-term rainfall averages: the occurrence of an extreme rainfall event in a given agricultural season is correlated with an increased likelihood of outmigration in the subsequent season [3]. In addition, the use of pesticides in China is very high, and China is the world’s biggest producer and consumer of pesticides; occupational, prenatal, and childhood exposure to pesticides has been associated with neurological impairment. Migration rates are statistically higher in areas of northern and northwestern China affected by desertification than in other areas [93], and areas with severe land degradation, such as the Loess Plateau, Inner Mongolia, and the Tarim Basin, are also impacted by desertification [94].

Migration does not take the environmental health risk away; it just moves it. Construction activity occurs in the spring and summer, when the largest number of seasonal migrant workers (SMWs) are in the country, and this activity is estimated to expose 50 million workers to silica dust (silicosis) exposure, noise-induced hearing loss (NIHL), and heat exposure [21]. Exposure to organic solvents, heavy metals, noise, and repetitive strain is common among manufacturing workers in the Pearl River and Yangtze River deltas, and peripheral nerve damage has been linked to solvent exposure in workers at Dongguan e-waste factories, with more than 40% of workers having more than 3 years of experience in the industry exhibiting noise-induced hearing loss [21]. Although the hukou system has been reformed since 2014, migrants’ access to local healthcare, schooling, and social insurance coverage is still limited, and informal workers are not sufficiently covered [12, 95]. In Guangdong, the proportion of construction migrants who were able to identify hazards associated with silica dust was less than 30%, and less than half were able to use the respirators provided [21].

### Cross-cutting and moderating factors

Gender, age, and socioeconomic status are important determinants of exposure and the ability to react to it through migration.

Rural women suffer more from indoor air pollution because they cook and do housework. They also face higher barriers to migration because of childcare responsibilities, while male migrants are engaged in agricultural and pesticide-related tasks in the countryside. Both children and older adults are more vulnerable to the effects of pollutants [37] and are less likely to migrate, as evidenced by a large body of literature on age-graded migration [96–107]. Higher-income households have more resources to move and may also have access to protective measures such as water filters, whereas lower-income households are less likely to have the resources to protect themselves locally or to move [2], which can be referred to as a poverty-environment trap; there is also some evidence regarding whether higher-income households are more likely to participate in welfare programs and whether migrants have social insurance coverage [108–110]. These factors do not act alone but are interrelated: A socioeconomic factor influences exposure and mobility; gender influences exposure through household roles; and age influences mobility directly (Table 1).

**TABLE 1:** Environmental health exposures, health outcomes, and migration patterns by vulnerable population group. Systematic review, China, 2001–2025.

Population group	Primary environmental exposures	Key health outcomes	Migration pattern and barriers
Women in rural settings	Indoor solid fuel smoke; pesticide residues; lead, cadmium	Anemia; adverse birth outcomes; chronic respiratory disease	High environmental burden; limited mobility due to caregiving responsibilities
Children under 5	PM2.5; lead; pesticides; contaminated water	Impaired lung development; neurotoxicity; stunted growth	Moved by caregivers during pollution peaks; otherwise remain in high-risk areas
Older adults in rural settings	Cold stress; indoor coal smoke; arsenic or fluoride in water	COPD; cardiovascular events; renal dysfunction	Lowest mobility; isolated in degraded areas when working-age adults migrate
Agricultural workers [18–44, 116]	Extreme heat during harvest; pesticide exposure; dust	Heat illness; chronic kidney disease; organophosphate poisoning	High mobility; seasonal circular migration between farming and construction or factory work
Construction migrants [18–49, 116]	Silica dust; extreme heat; noise; solvents	Silicosis; COPD; hearing loss; solvent neuropathy	Migrate to urban destinations; occupational exposures add to burdens from their places of origin
Pregnant women	Pesticides; heavy metals; air pollution	Preterm birth; low birth weight; neurodevelopment deficits in newborns	Some migration to escape pollution peaks pollution peaks; access to prenatal care often blocked by the hukou system

These five environmental domains also interact with each other and should not be examined individually. In the north, winter air pollution occurs in the same households as cold-exposure-related health risks; at the same time, in the same season, a combination of PM2.5 and heat stress during the summer season could be more harmful than either stressor individually. In rural areas, water scarcity is often coupled with insecure agriculture: when agricultural production is reduced due to drought, so is rural income, which in turn reduces water supply. In the places they travel to, individuals who already have health problems from the heavy metals or pesticides to which they were exposed in their own country may be exposed to more silica dust or chemicals at work sites or in factories. Community-level social capital, place attachment, and intergenerational solidarity also influence the experience and reaction of migrant populations to these combined exposures [111–115].

### Seasonal patterns of environmental stress

Air pollution is highest in winter when individuals are using home heating; heat stress is highest in the summer; water scarcity is highest in the spring before the monsoon season; pesticide exposure is highest from spring through summer, when crops are being planted and grown; and agricultural disruption and outmigration are highest in the fall and early winter, after the harvest season (Figure 5).

**FIGURE 5:**
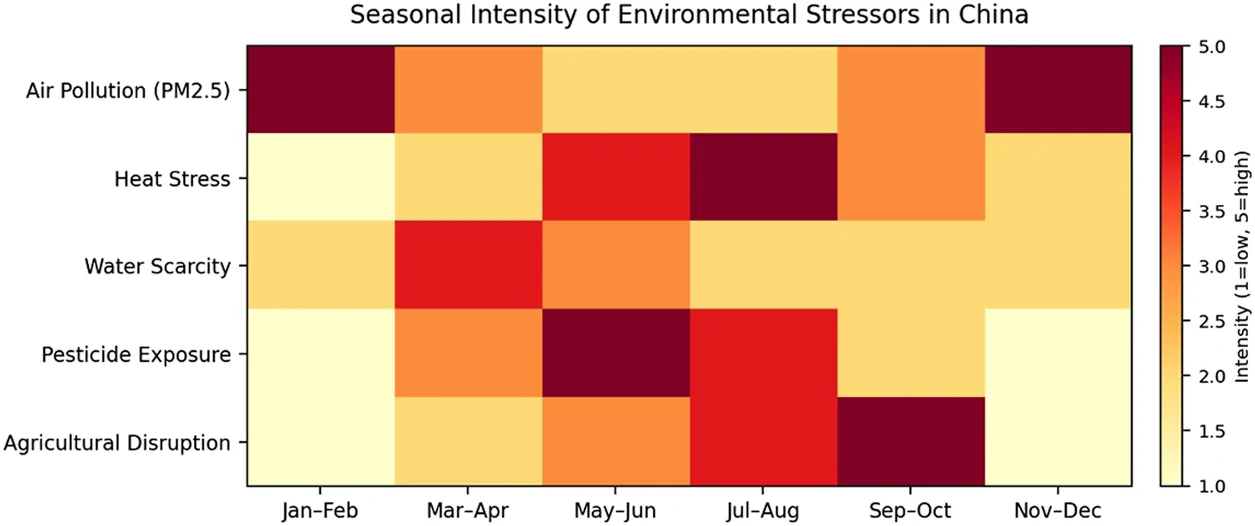
Seasonal intensity of environmental stressors in China, on a scale from 1 (low) to 5 (high). Systematic review, China, 2001–2025.

This results in an overall foreseeable migration pattern: outmigration in autumn after harvest, with increasing air pollution and decreasing demand for farm labor; return migration in spring when land is being prepared.

## Discussion

### Policy implications

Although there have been measurable improvements in some areas, such as the 2014 Air Pollution Action Plan, partial hukou reform, and the 2015 extension of pollution control to soil and water, environmental health governance for migrants is still underdeveloped, and the “Healthy China 2030” initiative lacks a focus on environmental migrants.

At the national level, there is potential to connect environmental monitoring data to migrant registration to monitor the health effects of environmental migration, and there is potential for more reform in the hukou system to enhance migrants’ access to healthcare, especially for occupational diseases. High pollution and climate-sensitive hotspots require increased occupational health safeguards at migration destinations, with mandatory health screening for construction workers, heat illness prevention regulations, and a wider spread of occupational health clinics, incentivizing construction workers in high-inflow provinces (including Guangdong, Zhejiang, Jiangsu, and Beijing). A seasonal response could be achieved at the community level through the use of low-cost air and water sensors, real-time digital air and water alerts, and culturally appropriate health education on topics such as air pollution, heat illness, pesticide safety, and water testing.

Specific evidence-based measures include coordinated air quality alerts during the November-March heating season in cities whose pollution levels are above 100 μg per cubic meter of PM2.5; subsidized respiratory screenings in high pollution areas; targeted risk communication in outsourcing communities about protective measures; mandatory rest breaks and heat protection rules for outdoor workers; climate-resilient agricultural support and crop insurance in high outmigration counties; and accelerated groundwater remediation with targeted blood lead screening in provinces with known contamination such as Hunan and Guangdong. These recommendations are based directly on the exposure pathways and populations listed above, and are more specific than policy goals, moving toward those interventions that can be implemented and evaluated.

### Limitations

There are some gaps in the current evidence base. Longitudinal, household-level studies of exposure, health, and migration over time are limited, with the majority of studies being cross-sectional and area-level, hence not able to isolate environmental factors from economic factors of migration at the individual level. These also cannot draw conclusions about causality. Environmental and environmental-economic motivations are also very often grouped in the same household decision, which makes the distinction between these motivations difficult to assess in the available data. Publication bias may further work against studies that do not report a clear environment-migration link. The definitions, exposure measures, and geographic scope differ extensively between studies, making direct comparisons difficult, and only a few studies measure seasonal migration as a primary outcome; rather, they extract its relevance from data gathered for other aims.

Due to the heterogeneity of study designs, exposure measures, and definitions of outcomes across the 116 sources included, a formal, standardized risk-of-bias assessment was not possible. This is recognized as a limitation that restricts the capacity for formal weighting of the underlying evidence’s relative strength; future domain-specific systematic reviews based on this framework should use an appropriate and structured tool for the study design–ROBINS-I for non-randomized studies, Cochrane RoB 2 for controlled trials, and the Newcastle-Ottawa Scale for cohort and case-control studies.

Other research priorities for future studies include the health effects that may result from combined and simultaneous exposures (e.g., combined exposure to PM2.5 and heat, or exposure to pesticides and contaminated water), which remain under-researched among Chinese migrant populations; the degree to which occupational risk factors faced at the destination site add to the health impact of the environmental exposure that was experienced at the point of origin; and the analysis of the motives for environmental migration and health impacts disaggregated by gender, since gender is clearly a determinant of exposure and the ability to migrate.

### Conclusion

This review synthesizes evidence from air quality, thermal stress, water pollution and soil pollution, agricultural instability, and occupational hazards to demonstrate that environmental conditions in China are a multi-dimensional, seasonally organized driver of population movement. Air pollution appears to influence out-migration from the north of China the most; thermal stress and agricultural disruption drive migration from farming areas; and heavy-metal contamination drives migration from industrial hotspots. Gender, age, and socio-economic factors always influence who is exposed and who can move. While there has been some progress in policy interventions, especially in combating urban air pollution, there has been no systematic attempt to address the environmental health aspects of seasonal migration specifically. Observed migration patterns are not caused by a single factor but by many factors that are determined by socioeconomic vulnerability, which means that integrated research approaches and policies will be needed to address them, and that there will be a need to connect environmental protection, migrant health services, and social equity. The first step in protecting individuals and the environments they traverse is acknowledging the importance of environmental health in the migration of the Chinese population.

This review adds to the literature by providing the first study to connect five environmental health domains of air quality, thermal stress, water and soil pollution, agricultural instability, and destination-site occupational stress to a seasonal calendar for seasonal migration in China, highlighting the times of year when each stressor is most likely to occur, who is most likely to be impacted, and where initiatives can be best coordinated. This framework explicitly considers occupational hazards, seasonality, and the floating population in China; the majority of previous analyses of the global climate-migration nexus have neglected such considerations [4, 19, 20].
